# Association between the Korean Healthy Diet Score and Metabolic Syndrome: Effectiveness and Optimal Cutoff of the Korean Healthy Diet Score

**DOI:** 10.3390/nu16193395

**Published:** 2024-10-06

**Authors:** Soo-Hyun Kim, Hyojee Joung

**Affiliations:** 1 Department of Public Health, Graduate School of Public Health, Seoul National University, Seoul 08826, Republic of Korea; kimsoo135@snu.ac.kr; 2 Institute of Health and Environment, Seoul National University, Seoul 08826, Republic of Korea

**Keywords:** healthy diet score, metabolic syndrome, cut off, Republic of Korea

## Abstract

Objectives: Although the prevalence of metabolic syndrome has increased among Koreans, the specific health benefits of the Korean Healthy Diet score remain unexplored. This study aimed to evaluate the association between the Korean Healthy Diet score and metabolic syndrome and to identify the optimal cutoff of the Korean Healthy Diet score for reducing metabolic syndrome prevalence. Methods: This cross-sectional study used data from 11,403 participants of the seventh and eighth Korea National Health and Nutrition Examination Survey. The Korean Healthy Diet score was calculated based on adherence to 13 dietary components. A logistic regression analysis was used to determine the association between the Korean Healthy Diet score and metabolic syndrome, as well as to identify the optimal cutoff values for the Korean Healthy Diet score. Results: The average Korean Healthy Diet score was significantly lower in participants with metabolic syndrome than in those without metabolic syndrome (5.03 vs. 5.14, *p* = 0.016). A one-point increase in the Korean Healthy Diet score was associated with a reduction in metabolic syndrome prevalence (odds ratio: 0.95, 95% confidence interval: 0.91–0.98). The optimal cutoff for the Korean Healthy Diet score was identified as >7 points, particularly showing significantly decreased prevalence of hypertriglyceridemia and low high-density lipoprotein cholesterol. Conclusions: The Korean Healthy Diet score was inversely associated with metabolic syndrome prevalence, and the identified optimal cutoff values can serve as a practical tool for public health interventions aimed at reducing the risk of metabolic syndrome.

## 1. Introduction

According to the National Health Insurance Service, the prevalence of metabolic syndrome (MetS) in Korea has steadily increased from 19.2% in 2019 to 22.0% in 2022 [1,2]. Among Koreans, 69.4% have at least one risk factor for MetS, and the prevalence of each MetS component is as follows: 25.7% for abdominal obesity, 45.4% for high blood pressure, 40.5% for hyperglycemia, 18.8% for hypertriglyceridemia, and 16.0% for low high-density lipoprotein (HDL) cholesterol [2]. This increasing prevalence can be attributed to dietary changes associated with the adoption of a Western lifestyle [3], wherein the consumption of refined grains and meat has increased, and the intake of dietary fiber, vegetables, and grains, which are the main components of the traditional Korean diet, has decreased, leading to an increased risk of MetS [4,5]. Aging and lifestyle changes have contributed to an increasing prevalence of MetS [6], which is known to increase the risk of developing diabetes by 30–52%, cardiovascular disease by 12–17%, and all-cause mortality by 6–7% [7]. Thus, the prevention and management of MetS is necessary, and dietary improvement plays a critical role in preventing MetS.

According to the American Heart Association, managing MetS requires adopting a diet rich in fruits, vegetables, whole grains, skinless poultry, fish, nuts, lean meats, and vegetable proteins, while limiting the intake of processed foods, saturated and trans fats, red meat, sodium, and added sugars [8]. However, over the past 20 years, the consumption of grains, vegetables, carbohydrates, sodium, and vitamin C among Koreans has decreased, while the intake of beverages, dairy products, meat, eggs, and fats has increased [9]. In addition, Koreans are not meeting the essential dietary factors stated in the Health Plan 2030, which aims to promote healthy eating and optimal nutritional status, suggested by the Ministry of Health and Welfare and Korea Health Promotion Institute [10]. According to the 2022 Korean Health Statistics, only 22.7% of Koreans consume more than 500 g of fruits and vegetables per day, 43.8% consume saturated fats at an appropriate level, and only 35.5% meet the recommended sodium intake [10]. Furthermore, only 14.1% and 14.5% of individuals meet the adequate intake levels of calcium and vitamin A, respectively, while the proportion of individuals practicing a healthy diet defined by the Health Plan 2030 is 43.7% [10].

To prevent MetS more effectively, there has been a shift from the previous approach that focused solely on individual foods or nutrients toward dietary patterns that better reflect the overall dietary quality of the population [11,12]. Recent studies have also reported that intervention with a healthy diet is more effective in preventing diseases than restricting calories or specific nutrients [13,14,15]. Many researchers have developed various dietary indices to assess the diet quality in Korea [16,17,18,19,20]. Indices adapted from international models to better reflect the dietary habits of the Korean population include the Korean Healthy Eating Index (KHEI) and Korean Diet Quality Index [16,17]. Other indices, such as the Korean Dietary Pattern Score, Korean Diet Score, and Nutrition Quotient for Adults (NQ), have been developed for specific research purposes [18,19,20]. However, with a few exceptions, most of these indices have not been regularly updated, which limits their ability to accurately reflect current dietary changes. The KHEI, one of the most widely used indices in Korea, offers a detailed evaluation of vegetable and fruit intake by dividing the total vegetables into subcategories, such as vegetables excluding kimchi, and total fruits into fresh fruits [16]. Although this provides a more detailed assessment of vegetable and fruit intake, it also introduces complexity into the classification process. Additionally, the sodium intake evaluation of the KHEI does not reflect the newly introduced concept of chronic disease risk reduction intake from the 2020 Korean Dietary Reference Intakes (KDRIs). The widely used NQ is a simplified assessment tool that individuals can complete without expert dietary assessment [20]. Although it offers the advantage of being a simple way to evaluate dietary quality, it may be less accurate in determining the actual intake levels and is subject to the limitations of self-reporting [21]. The Korean Healthy Diet (KHD) score, recently developed to promote health and prevent diseases among Koreans, includes eight nutrients (carbohydrates, sugar, dietary fiber, protein, fat, saturated fat, sodium, and calcium) and five food groups (whole grains, meat/fish/eggs/legumes, vegetables, fruits, and dairy products) [22]. The scoring system was based on the 2020 KDRIs and target patterns for different sex and age subgroups [23]. The KHD score improves upon previous Korean dietary indices by reflecting the latest 2020 KDRIs, using objective data for diet quality evaluation, and offering a simplified binary scoring system, making the evaluation process easier.

Although the KHD score was developed to enhance health and prevent diseases in Koreans, the specific health benefits associated with adherence to the KHD have not been investigated [22]. Categorizing adherence levels to the KHD using cutoff points simplified the scores to make health recommendations for the general population and health professionals more practical. This approach provides clearer guidance for dietary improvements, supports better comparisons across populations, and helps monitor changes in dietary behavior over time [24]. Therefore, this study aimed to investigate the association between the KHD score and its components with MetS, and to suggest the optimal cutoff values of the KHD score related to MetS risk among Korean adults using data from the seventh and eighth Korea National Health and Nutrition Examination Survey (KNHANES; 2016–2021).

## 2. Materials and Methods

### 2.1. Study Subjects

This study utilized data from the 7th and 8th KNHANES conducted by the Korea Centers for Disease Control and Prevention from 2016 to 2021. The KNHANES is a nationally representative cross-sectional survey that employs a stratified, multistage, clustered probability sampling design to assess the overall lifestyle of a representative sample of Koreans. The original data are publicly accessible on the KNHANES website (http://knhanes.cdc.go.kr), and additional details of the study design and protocols have been described in previous reports and websites [25]. For this study, raw data from the KNHANES were obtained on 30 May 2024, and the study protocols were approved by the Institutional Review Board of the Seoul National University (IRB No. 2209/003-001).

Among the 46,828 individuals who participated in the 7th and 8th KNHANES, we excluded 29,747 participants aged < 19 or >49 years; 922 participants with missing information required for complex sample analysis; 2794 participants with missing data in their 24 h dietary record; 407 participants with a fasting time of less than 8 h; 103 participants who were pregnant; 737 participants with hypertension, diabetes, or dyslipidemia; 260 participants who reported implausible daily energy intakes (<500 kcal or >5000 kcal per day); and 455 participants with missing baseline information. Of the total, 11,403 participants were included in the final sample.

### 2.2. Variables

MetS was diagnosed based on the criteria established by the National Cholesterol Education Program Adult Treatment Panel III [26], which was defined as the presence of at least three of the following metabolic components: hyperglycemia (fasting glucose ≥ 100 mg/dL or currently taking medication for diabetes), hypertriglyceridemia (triglycerides ≥ 150 mg/dL), low HDL cholesterol (<40 mg/dL in men; <50 mg/dL in women), high blood pressure (systolic blood pressure ≥ 130 mmHg or diastolic blood pressure ≥ 85 mmHg), or abdominal obesity (waist circumference > 90 cm in men or >85 cm in women). For waist circumference, the criteria of the Korean Society for the Study of Obesity were applied [27].

Overall diet quality was assessed using the KHD score, which has been described in a previous study [22]. The KHD score was calculated using dietary survey data obtained through the 24 h recall method from the KNHANES. Participants who met the criteria for each KHD component were assigned 1 point, whereas those who did not were assigned 0 points. Therefore, the total KHD score ranged from 0 to 13, with a higher score indicating greater adherence to the KHD.

The characteristics of the participants in this study were based on data from the KNHANES, which included sex, age (grouped into 19–29 and 30–49 years), body mass index (BMI; underweight, normal, and overweight), smoking status (current or not), alcohol consumption (more than once a month or not), physical activity, defined as engaging in moderate- or high-intensity physical activity (yes or no), household income level (low or high), education level (over college or not), and household status (single or multi-person).

### 2.3. Statistical Analysis

Statistical analysis was performed using SAS software (version 9.4; SAS Institute Inc., Cary, NC, USA), with significance set at a two-tailed *p*-value of <0.05. Given that the KNHANES data reflect a complex sampling design, all statistical analyses were performed using the PROC SURVEY procedure, which is appropriate for complex sample analyses. Descriptive statistics were calculated to assess general characteristics according to MetS status, using independent *t*-tests and chi-square tests to identify significant differences. Subsequently, independent *t*-tests and logistic regression analyses were conducted to examine the differences in adherence to each KHD component according to MetS status. Further, logistic regression analyses were performed to identify the odds of MetS according to the KHD score and its various categories to determine the optimal cutoff for reducing MetS prevalence. The KHD score was categorized into quartiles, tertiles, and sequential classifications based on the score ranges. Finally, using the optimal cutoff determined from the results, the relationship between the optimal KHD score category and MetS components was examined. All logistic regression analyses were adjusted for sex, age, smoking status, alcohol consumption, physical activity, household income, educational level, and household status.

## 3. Results

### 3.1. Characteristics of the Study Subjects by MetS Status

The characteristics of the study participants stratified by MetS status are presented in Table 1. A total of 11,403 participants were included in the analysis, of whom 1668 (14.63%) had MetS. Approximately 51.5% of the participants were female and 67.1% were 30–49 years. Participants with MetS were significantly older, more likely to be men, had an abnormal BMI, were more likely to be current smokers, consumed more alcohol, were less physically active, had lower household incomes, and were less educated than those without MetS (all *p* < 0.05).

The clinical status of the subjects indicated that, among the MetS components, the highest prevalence rates were observed in the following order: hypertriglyceridemia, abdominal obesity, low HDL cholesterol, hyperglycemia, and high blood pressure. Hypertriglyceridemia was the most prevalent component in individuals with MetS, whereas low HDL cholesterol was the most prevalent component in individuals without MetS.

### 3.2. Relationship between Adherence to the KHD Components and MetS

Table 2 shows the relationship between adherence to the KHD score and its components and the prevalence of MetS. The average KHD score was 5.13 ± 0.02, and those with MetS had a significantly lower KHD score (5.03 ± 0.04) compared to those without MetS (5.14 ± 0.02, *p* = 0.016). The highest adherence was observed for sugar (86.0%) and protein (85.8%), whereas the lowest adherence was observed for calcium (15.2%), fruits (16.9%), and mixed grains (17.6%). Components with adherence rates below 50% included carbohydrates (32.6%); fiber (37.0%); saturated fat (48.1%); sodium (29.4%); meat, fish, eggs, and beans (34.9%); vegetables (20.1%); and milk and milk products (29.3%). The adherence rates were significantly lower for fiber (32.4%), sodium (22.1%), fruit (11.3%), and milk and milk products (23.5%) in the MetS group than in the non-MetS group (37.8%, 30.6%, 17.9%, and 30.3%, respectively; all *p* < 0.05). However, the adherence rates for sugar (88.4%), saturated fat (54.5%), and vegetables (27.0%) were significantly higher in the MetS group than in the non-MetS group (85.6%, 47.0%, and 18.9%, respectively; all *p* < 0.05). The prevalence of MetS was significantly lower in participants with higher KHD scores and a greater adherence to fiber (odds ratio [OR]: 0.82, 95% confidence interval [CI]: 0.72–0.94), fruits (OR: 0.67, 95% CI: 0.55–0.81), and milk and milk products (OR: 0.83, 95% CI: 0.72–0.95) after adjusting for sex, age, smoking status, alcohol consumption, physical activity, household income, education level, and household status.

### 3.3. Exploring the Optimal KHD Score Cutoff Related to MetS

The relationship between the KHD score and the prevalence of MetS, along with the results of determining the optimal KHD score cutoff to reduce the odds of MetS, are presented in Table 3. The results showed that each one-point and two-point increase in the KHD score was significantly associated with a reduced prevalence of MetS (one-point, OR: 0.95, 95% CI: 0.91–0.98; two-point, OR: 0.90, 95% CI: 0.84–0.97) after adjusting for sex, age, smoking status, alcohol consumption, physical activity, household income level, education level, and household status.

To identify the optimal cutoff for the KHD score, the score was divided into quartiles, tertiles, and sequential classifications by score range. In the quartile division, the scores were categorized as follows: 1–3 points for Q1, 4–5 points for Q2, 6 points for Q3, and 7–13 points for Q4. Participants in the highest quartile (Q4) had significantly lower odds of developing MetS than participants in the lowest quartile (Q1; OR: 0.77, 95% CI: 0.63–0.95). However, this effect was not observed when the participants were stratified by sex. When the scores were divided into tertiles, the categories were 1–4 points for T1, 5 points for T2, and 6–13 points for T3. A significant reduction in odds was observed for women (OR: 0.77, 95% CI: 0.61–0.97), while no significant reduction was found for the total participants or men.

When the KHD score was divided into three groups, various cutoff versions were tested: version 1 (1–3, 5–6, 7–13 points), version 2 (1–4, 5–7, 8–13 points), and version 3 (1–4, 5–8, 9–13 points). In the total participants, all three versions showed a significant reduction in the odds of MetS in the high category compared to the low category (version 1, OR: 0.78, 95% CI: 0.65–0.93; version 2, OR: 0.72, 95% CI: 0.56–0.91; version 3, OR: 0.67, 95% CI: 0.46–0.99). When stratified by sex, women showed significantly lower odds for all three versions, whereas no significant effect was observed in men. Further analysis was conducted by dividing the KHD score into two groups and testing the cutoff values from five to nine points. As the cutoff value increased, the odds of having MetS decreased across all participants (OR: 0.89, 95% CI: 0.78–1.02 for >5 points; OR: 0.88, 95% CI: 0.77–1.01 for >6 points; OR: 0.80, 95% CI: 0.68–0.94 for >7 points; OR: 0.75, 95% CI: 0.60–0.94 for 8 points; OR: 0.72, 95% CI: 0.49–1.04 for 9 points). When stratified by sex, the odds for MetS had significant reductions for women at five points (OR: 0.75, 95% CI: 0.61–0.92) and for men at seven points (OR: 0.78, 95% CI: 0.63–0.96).

### 3.4. Relationship between the Optimal KHD Score Cutoff and MetS Components

The association between the optimal KHD score cutoff (categorized as low, medium, and high; 1–4, 5–6, and 7–13 points, respectively, as determined from the results in Table 3) and the MetS components is presented in Table 4. There were significantly reduced odds in the groups who scored high compared to those who scored low for hypertriglyceridemia (OR: 0.80, 95% CI: 0.69–0.94, *p*-trend = 0.009) and HDL cholesterol (OR: 0.85, 95% CI: 0.74–0.98, *p*-trend = 0.027) in the total participants after adjusting for sex, age, smoking status, alcohol consumption, physical activity, household income level, education level, and household status. When stratified by sex, women in the group who scored high had significantly lower odds of hypertriglyceridemia compared to the group who scored low (OR: 0.76, 95% CI: 0.60–0.96, *p*-trend = 0.016).

## 4. Discussion

This study was conducted to evaluate the association between the KHD score and MetS, as well as to determine the optimal KHD score cutoff among Koreans. A higher adherence to the KHD score and its components, such as fiber, fruits, and milk and milk products, were significantly associated with a decreased prevalence of MetS. Furthermore, the optimal cutoff of the KHD score predicting a lower prevalence of MetS was identified as >7 points out of the total 13 points.

The KHD score, which assesses diet quality by summing the adherence scores of each KHD component, has been proven to be a strong predictor of MetS prevalence. In this study, individuals without MetS had significantly higher KHD scores, and higher KHD scores were associated with lower odds of developing MetS, indicating that a greater adherence to KHD is related to a reduced risk of MetS. Previous studies on healthy diet scores such as the Mediterranean Diet Score, Healthy Eating Index, and Dietary Approaches to Stop Hypertension have consistently demonstrated that higher scores, which reflect a better adherence to healthy dietary patterns, were associated with a lower risk of MetS, cardiovascular diseases, and other metabolic disorders [28]. Consistent with international research on the beneficial impact of diet on preventing MetS and other diseases, the KHD score serves as a meaningful tool for reducing the risk of MetS.

Dietary fiber, fruits, milk, and milk products were significantly associated with MetS. An increased intake of these components has been associated with improvements in glucose tolerance, reductions in LDL cholesterol, and decreases in waist circumference, as distinguished in previous studies [29,30,31,32,33,34,35]. However, despite the recognized importance of these components, their adherence rates were lower than those of other KHD components, highlighting a trend in low intake levels. In contrast, for other KHD components, such as sugar, saturated fat, and vegetables, higher adherence was observed in participants with MetS, but there was no significant reduction in the prevalence of MetS. This outcome is consistent with several studies based on KNHANES data, which also found no significant association between the intake of sugar, saturated fat, and vegetables and MetS prevalence [36,37,38]. Nevertheless, it is important to exercise caution regarding the intake of these components because the excessive consumption of sugar and saturated fat has been associated with a plasma cholesterol level and an increased risk of MetS in numerous studies [29,39,40]. Additionally, a previous study revealed an inverse association between vegetable consumption and MetS risk in Asians, and these discrepancies may be explained by the form in which vegetables are consumed [33]. In Korea, approximately 40% of the total vegetable intake comes from kimchi, a fermented vegetable high in sodium, which has been associated with a higher prevalence of MetS [41]. However, after adjusting for sodium intake, kimchi consumption did not negatively affect the development of MetS. Furthermore, other KHD components such as carbohydrates, protein, fat, sodium, calcium, mixed grains, meat, fish, eggs, and beans are important for maintaining health and preventing diseases by controlling blood pressure and plasma lipid levels [29,41,42,43,44,45,46]. Therefore, it is essential to emphasize adherence to all components of the KHD to effectively reduce risk factors of MetS and promote overall health.

Composing an index of diet quality involves several considerations, including the number of components to be included, scoring criteria, and their respective cutoff values to define an optimal diet [47]. Using scientifically grounded cutoff points, such as those in dietary guidelines, may be a valuable tool for comparing diet quality [48]. A previous study emphasized that using cutoff values in simplified dietary assessments can effectively identify individuals at risk, thereby supporting targeted nutritional interventions [49]. The 13-point KHD score is a simple and effective way to promote healthier eating habits and help individuals understand and follow a healthy diet. The observed reduction in MetS prevalence with each one- and two-point increase in the KHD score confirmed its effectiveness. However, to enhance the usability of the KHD score, it is essential to establish cutoff values that make it easier for the general public to understand and apply.

To determine the ideal cutoff value, we examined various cutoff points and their association with the prevalence of MetS. The results showed that as the cutoff values for the KHD score increased, the odds of MetS decreased. In addition, when dividing the KHD score into quartiles, tertiles, and sequential classifications by score ranges, the highest category showed significantly lower odds of MetS than the lowest category across quartiles and several sequential classifications. However, within the quartiles (1–3, 4–5, 6, and 7–13 points), a cutoff point was identified at 6 points for Q3. Despite the large proportion of participants falling within this range, the distribution of scores was too narrow. Moreover, when categorizing participants with a high score of ≥8 points, the number of participants in the group was too small, representing less than 10% of the total participants. Considering these statistically significant results and the overall distribution of participants, it seems reasonable to set the high-adherence group at seven points. Since dividing the KHD score into three groups using version 1 (1–4, 5–6, and 7–13 points) showed significant differences in the odds of MetS, this was determined to be the optimal cutoff value. This approach is supported by previous studies on Mediterranean diet scores, which typically calculate diet scores by summing the binary adherence scores of components, similar to the KHD scoring method, and often categorize adherence scores into three levels: low (0–3 points), medium (4–5 or 6 points), and high (6 or 7–9 points), as defined by researchers based on the distribution of the subjects and their scores [50,51].

The prevalence of MetS is significantly influenced by poor dietary quality and unhealthy lifestyle behaviors, and numerous studies have established a strong association between MetS and various lifestyle factors [52,53]. Among these factors, modifiable lifestyle behaviors, such as smoking, alcohol consumption, and diet quality, have a major impact, and targeting these behaviors through intervention and education can be effective for prevention [54]. Previous research has indicated that individuals who are less educated are more likely to develop MetS, particularly at an earlier stage in their lives, than those who are highly educated; hence, education regarding various lifestyle factors is essential for the prevention of MetS. In Korea, tools such as the KDRIs and target patterns are commonly used in nutrition education. The KDRIs provide guidelines on dietary practices to promote the health of the Korean population, and the target pattern based on the KDRIs outlines the recommended levels of food intake [23]. Adherence to these guidelines reduces the prevalence of MetS [46,55]. While the KDRIs are difficult to understand and lack easily applicable solutions [56], the KHD score is designed to be simple for the general public to understand and evaluate. Therefore, the KHD score, which is based on the KDRIs and target patterns with a simplified evaluation process, could be an effective tool for nutritional education aimed at preventing MetS.

The prevalence of MetS in Korea has been steadily increasing, and an analysis of MetS component prevalence in 2021 revealed that the prevalence of abdominal obesity and high blood pressure decreased by 0.4% compared with the previous year, while the prevalence of high blood sugar remained relatively unchanged [2]. In contrast, the prevalence of hypertriglyceridemia and low HDL cholesterol increased significantly by 5.9% and 6.0%, respectively, highlighting the urgent need to manage triglyceride and HDL cholesterol levels among Koreans. In this context, the findings of this study, which show that higher optimal KHD scores are associated with a lower prevalence of hypertriglyceridemia and lower HDL cholesterol levels, further validate the effectiveness of the KHD score in managing MetS among Koreans.

The present study has several limitations. First, the KNHANES provides a representative sample of the entire Korean population, but the inherent nature of a cross-sectional study limits the ability to determine causal relationships or the exact risk reduction of MetS. Although this cross-sectional design allowed for a broad assessment of the relationship between the KHD score and MetS prevalence, future research should explore the incidence of MetS over time and conduct large-scale trials in diverse populations to strengthen the evidence. Second, the use of a single 24 h recall diet information may not reflect individuals’ usual nutrient intakes, even though a skilled technician conducted it to minimize measurement errors. To better assess the relationship between diet and disease risk, future studies need to use sufficient multiple-day data collection for estimating usual diet rather than a single-day intake. In addition, the analysis was retrospective, which introduced the potential for methodological bias. To enhance the utility of the KHD score, the KHD criteria should be applicable to broader populations. Thus, future studies should explore its association with other chronic diseases, such as cardiovascular disease, type 2 diabetes, and cancer, to examine its broader health impacts in various populations. Despite these limitations, the present study has the significant strength of being the first study to evaluate the association between KHD adherence and MetS. These findings highlight that a healthy diet is advantageous in reducing the prevalence of MetS, as evidenced by a large population study. Identifying the optimal cutoff for lowering the prevalence of MetS could be clinically useful for its prevention and underscores the potential of the KHD score as a valuable tool for assessing diet quality.

## 5. Conclusions

This study demonstrated that the KHD score and its optimal cutoff are potential tools for managing MetS. These findings further support the use of the KHD score to estimate diet quality in epidemiological research and nutrition management programs, thereby contributing to the promotion of health and disease prevention among Koreans.

## Figures and Tables

**Table 1:** Characteristics of the participants according to MetS.

	Total (*n* = 11,403)	MetS (*n* = 1668)	Non-MetS (*n* = 9735)	*p*-Value
Women, *n* (%)	6680 (51.5)	593 (28.6)	6087 (55.6)	<0.001
30–49 years, *n* (%)	8234 (67.1)	1432 (83.0)	6802 (64.3)	<0.001
BMI (18.5–24.9 kg/m^2^), *n* (%)	7195 (61.7)	312 (18.0)	6883 (69.5)	<0.001
Current smokers, *n* (%)	2185 (21.4)	515 (32.8)	1670 (19.3)	<0.001
Alcohol consumption, over once a month, *n* (%)	7292 (65.0)	1125 (67.9)	6167 (64.5)	0.018
Physical activity (yes), *n* (%)	5751 (52.6)	751 (46.7)	5000 (53.7)	<0.001
Household income level (high), *n* (%)	8034 (70.1)	1109 (67.4)	6925 (70.6)	0.025
Education level (over college), *n* (%)	6904 (59.1)	931 (55.7)	5973 (59.7)	0.011
Household status (single), *n* (%)	993 (9.5)	153 (10.1)	840 (9.4)	0.409
Abdominal obesity, *n* (%)	2595 (23.6)	1298 (78.8)	1297 (13.9)	<0.001
High blood pressure, *n* (%)	1905 (17.5)	959 (58.4)	946 (10.3)	<0.001
Hyperglycemia, *n* (%)	2287 (19.8)	1128 (67.3)	1159 (11.4)	<0.001
Hypertriglyceridemia, *n* (%)	2608 (23.8)	1343 (80.5)	1265 (13.8)	<0.001
Low HDL-cholesterol, *n* (%)	2777 (23.4)	1078 (63.1)	1699 (16.4)	<0.001

Values are presented as *n* (%) and *p*-values were calculated using the chi-square test. Participant characteristics were classified based on Korea National Health and Nutrition Examination Survey data: age groups (19–29 years, 30–49 years); BMI (<18.5 kg/m^2^, 18.5–24.9 kg/m^2^, ≥25.0 kg/m^2^); smoking status (current smoker or not); alcohol consumption (over once a month or not); physical activity defined as engaging in moderate-intensity physical activity for at least 150 min per week, high-intensity physical activity for at least 75 min per week, or an equivalent combination of both, where 1 min of high-intensity activity is equivalent to 2 min of moderate-intensity activity (yes or no); household income level (low, high); education level (college or higher, less than college); and household status (single or not). MetS, metabolic syndrome; BMI, body mass index; HDL, high-density lipoprotein.

**Table 2:** Relationship between adherence to the KHD and MetS.

KHD Score and Its Components	Total	MetS	Non-MetS	*p*-Value	OR (95% CI)
KHD score	5.13 (0.02)	5.03 (0.04)	5.14 (0.02)	0.016	0.95 (0.91–0.98)
Carbohydrate	3704 (32.6)	515 (31.4)	3189 (32.8)	0.299	0.96 (0.84–1.10)
Protein	9850 (85.8)	1454 (86.6)	8396 (85.7)	0.401	0.95 (0.79–1.14)
Fat	6770 (59.6)	958 (58.3)	5812 (59.8)	0.329	0.91 (0.80–1.04)
Fiber	4512 (37.0)	591 (32.4)	3921 (37.8)	<0.001	0.82 (0.72–0.94)
Sugar	9788 (86.0)	1475 (88.4)	8313 (85.6)	0.005	1.12 (0.93–1.35)
Saturated fat	5550 (48.1)	915 (54.5)	4635 (47.0)	<0.001	1.10 (0.97–1.25)
Sodium	3420 (29.4)	381 (22.1)	3039 (30.6)	<0.001	0.92 (0.79–1.07)
Calcium	1777 (15.2)	238 (14.1)	1539 (15.4)	0.225	0.87 (0.73–1.04)
Mixed grains	2035 (17.6)	295 (17.4)	1740 (17.7)	0.832	0.93 (0.79–1.10)
Meat, fish, eggs, and beans	3954 (34.9)	593 (35.8)	3361 (34.8)	0.475	1.03 (0.90–1.18)
Vegetables	2321 (20.1)	450 (27.0)	1871 (18.9)	<0.001	1.12 (0.96–1.29)
Fruits	2154 (16.9)	215 (11.3)	1939 (17.9)	<0.001	0.67 (0.55–0.81)
Milk and milk products	3301 (29.3)	387 (23.5)	2914 (30.3)	<0.001	0.83 (0.72–0.95)

Values are presented as mean (standard deviation) for the KHD score (total score of 13) and as *n* (%) for the KHD components, representing the number of participants who adhered to each criterion. *p*-values were calculated using independent *t*-tests for continuous variables and chi-square tests for categorical variables according to the prevalence of MetS. The ORs for MetS were adjusted for sex (men, women), age, smoking status (current or not), alcohol consumption (over once a month or not), physical activity, defined as engaging in moderate-intensity physical activity for at least 150 min per week, high-intensity physical activity for at least 75 min per week, or an equivalent combination of both, where 1 min of high-intensity activity is equivalent to 2 min of moderate-intensity activity (yes or no), household income level (low, high), education level (college or higher, less than college), and household status (single or not). KHD, Korean Healthy Diet; MetS, metabolic syndrome; OR, odds ratio; CI, confidence interval.

**Table 3:** Adherence to the Korean Healthy Diet according to metabolic syndrome prevalence, total participants, and sex.

		Total	Men	Women
	*n*	OR (95% CI)	OR (95% CI)	OR (95% CI)
Per 1-point increase	11,403	0.95 (0.91–0.98)	0.96 (0.91–1.00)	0.93 (0.87–0.99)
Per 2-point increase	11,403	0.90 (0.84–0.97)	0.92 (0.84–1.01)	0.87 (0.77–0.98)
Quartile				
Q1 (1–3)	1704	1.00	1.00	1.00
Q2 (4–5)	5279	0.96 (0.80–1.14)	0.99 (0.80–1.23)	0.87 (0.64–1.19)
Q3 (6)	2030	0.95 (0.76–1.18)	1.00 (0.76–1.31)	0.84 (0.58–1.22)
Q4 (7–13)	2390	0.77 (0.63–0.95)	0.78 (0.60–1.01)	0.74 (0.52–1.07)
Tertile				
T1 (1–4)	4181	1.00	1.00	1.00
T2 (5)	2802	0.94 (0.80–1.10)	1.07 (0.88–1.31)	0.71 (0.55–0.93)
T3 (6–13)	4420	0.86 (0.74–1.00)	0.91 (0.75–1.10)	0.77 (0.61–0.97)
Per category increase (version 1)				
Low (1–4)	4181	1.00	1.00	1.00
Medium (5–6)	4832	0.95 (0.82–1.09)	1.06 (0.88–1.26)	0.76 (0.61–0.95)
High (7–13)	2390	0.78 (0.65–0.93)	0.80 (0.64–1.01)	0.73 (0.54–0.97)
Per category increase (version 2)				
Low (1–4)	4181	1.00	1.00	1.00
Medium (5–7)	6187	0.92 (0.81–1.05)	1.01 (0.85–1.19)	0.77 (0.63–0.96)
High (8–13)	1035	0.72 (0.56–0.91)	0.79 (0.58–1.07)	0.60 (0.40–0.90)
Per category increase (version 3)				
Low (1–4)	4181	1.00	1.00	1.00
Medium (5–8)	6841	0.90 (0.79–1.03)	0.99 (0.84–1.17)	0.75 (0.61–0.93)
High (9–13)	381	0.67 (0.46–0.99)	0.67 (0.39–1.15)	0.68 (0.39–1.19)
Low vs. medium/high adherence				
1–4	4181	1.00	1.00	1.00
5–13	7222	0.89 (0.78–1.02)	0.97 (0.83–1.15)	0.75 (0.61–0.92)
Low vs. medium/high adherence				
1–5	6983	1.00	1.00	1.00
6–13	4420	0.88 (0.77–1.01)	0.88 (0.74–1.05)	0.88 (0.72–1.09)
Low/medium vs. high adherence				
1–6	9013	1.00	1.00	1.00
7–13	2390	0.80 (0.68–0.94)	0.78 (0.63–0.96)	0.84 (0.65–1.09)
Low/medium vs. high adherence				
1–7	10,368	1.00	1.00	1.00
8–13	1035	0.75 (0.60–0.94)	0.78 (0.58–1.05)	0.71 (0.48–1.03)
Low/medium vs. high adherence				
1–8	11,022	1.00	1.00	1.00
9–13	381	0.72 (0.49–1.04)	0.67 (0.40–1.14)	0.82 (0.48–1.40)

Values are presented as OR (95% CI) after adjusting for sex (men, women), age, smoking status (current or not), alcohol consumption (over once a month or not), physical activity, defined as engaging in moderate-intensity physical activity for at least 150 min per week, high-intensity physical activity for at least 75 min per week, or an equivalent combination of both, where 1 min of high-intensity activity is equivalent to 2 min of moderate-intensity activity (yes or no), household income level (low, high), education level (college or higher, less than college), and household status (single or not). OR, odds ratio; CI, confidence interval.

**Table 4:** OR of metabolic syndrome components by adherence level to the Korean Healthy Diet.

Score Range	Low	Medium	High	*p*-Trend
	1–4	5–6	7–13	
Abdominal obesity				
All participants	1.00	0.94 (0.84–1.06)	0.94 (0.82–1.08)	0.301
Men	1.00	0.98 (0.84–1.15)	0.90 (0.74–1.09)	0.316
Women	1.00	0.91 (0.76–1.08)	1.00 (0.81–1.23)	0.848
High blood pressure				
All participants	1.00	1.03 (0.91–1.18)	0.92 (0.77–1.09)	0.447
Men	1.00	1.10 (0.94–1.30)	0.96 (0.77–1.19)	0.995
Women	1.00	0.89 (0.71–1.11)	0.83 (0.63–1.09)	0.159
Hyperglycemia				
All participants	1.00	1.00 (0.88–1.13)	0.98 (0.83–1.15)	0.812
Men	1.00	1.03 (0.86–1.22)	1.03 (0.82–1.29)	0.780
Women	1.00	0.97 (0.81–1.17)	0.95 (0.75–1.20)	0.648
Hypertriglyceridemia				
All participants	1.00	0.96 (0.85–1.08)	0.80 (0.69–0.94)	0.009
Men	1.00	1.04 (0.89–1.20)	0.82 (0.67–1.00)	0.120
Women	1.00	0.83 (0.69–1.00)	0.76 (0.60–0.96)	0.016
Low HDL cholesterol				
All participants	1.00	0.94 (0.84–1.06)	0.85 (0.74–0.98)	0.027
Men	1.00	1.04 (0.87–1.24)	0.80 (0.63–1.01)	0.140
Women	1.00	0.87 (0.75–1.00)	0.87 (0.73–1.03)	0.076

Values are presented as OR (95% CI) after adjusting for sex (men, women), age, smoking status (current or not), alcohol consumption (over once a month or not), physical activity, defined as engaging in moderate-intensity physical activity for at least 150 min per week, high-intensity physical activity for at least 75 min per week, or an equivalent combination of both, where 1 min of high-intensity activity is equivalent to 2 min of moderate-intensity activity (yes or no), household income level (low, high), education level (college or higher, less than college), and household status (single or not). OR, odds ratio; CI, confidence interval; HDL, high-density lipoprotein.

## Data Availability

The data presented in this study are available from the second author upon request.
